# Isolation of Ultra-Small *Opitutaceae*-Affiliated Verrucomicrobia from a Methane-Fed Bioreactor

**DOI:** 10.3390/microorganisms13081922

**Published:** 2025-08-17

**Authors:** Olga V. Danilova, Varvara D. Salova, Igor Y. Oshkin, Daniil G. Naumoff, Anastasia A. Ivanova, Natalia E. Suzina, Svetlana N. Dedysh

**Affiliations:** 1 Winogradsky Institute of Microbiology, Research Center of Biotechnology of the Russian Academy of Sciences, Leninsky Ave. 33/2, Moscow 119071, Russia; salovavd@gmail.com (V.D.S.); ig.owkin@gmail.com (I.Y.O.); daniil_naumoff@yahoo.com (D.G.N.); ivanovastasja@gmail.com (A.A.I.); dedysh@mail.ru (S.N.D.); 2 Institute of Biochemistry and Physiology of Microorganisms, Pushchino Scientific Center for Biological Research of the Russian Academy of Sciences, Pushchino 142290, Moscow Region, Russia; suzina_nataliya@rambler.ru

**Keywords:** *Verrucomicrobiota*, *Opitutaceae* family, *Oleiharenicola* genus, ultra-small bacteria, methane-fed bioreactor, co-culture, heteropolysaccharide degradation, CAZymes

## Abstract

The bacterial phylum *Verrucomicrobiota* accommodates free-living and symbiotic microorganisms, which inhabit a wide range of environments and specialize in polysaccharide degradation. Due to difficulties in cultivation, much of the currently available knowledge about these bacteria originated from cultivation-independent studies. A phylogenetic clade defined by the free-living bacterium from oilsands tailings pond, *Oleiharenicola alkalitolerans*, and the symbiont of the tunicate *Lissoclinum* sp., *Candidatus* Didemniditutus mandelae, is a poorly studied verrucomicrobial group. This clade includes two dozen methagenome-assembled genomes (MAGs) retrieved from aquatic and soil habitats all over the world. A new member of this clade, strain Vm1, was isolated from a methane-fed laboratory bioreactor with a *Methylococcus*-dominated methane-oxidizing consortium and characterized in this study. Strain Vm1 was represented by ultra-small, motile cocci with a mean diameter of 0.4 µm that grew in oxic and micro-oxic conditions at temperatures between 20 and 42 °C. Stable development of strain Vm1 in a co-culture with *Methylococcus* was due to the ability to utilize organic acids excreted by the methanotroph and its exopolysaccharides. The finished genome of strain Vm1 was 4.8 Mb in size and contained about 4200 predicted protein-coding sequences, including a wide repertoire of CAZyme-encoding genes. Among these CAZymes, two proteins presumably responsible for xylan and arabinan degradation, were encoded in several MAGs of Vm1-related free-living verrucomicrobia, thus offering an insight into the reasons behind wide distribution of these bacteria in the environment. Apparently, many representatives of the *Oleiharenicola*–*Candidatus* Didemniditutus clade may occur in nature in trophic associations with methanotrophic bacteria, thus participating in the cycling of methane-derived carbon.

## 1. Introduction

*Verrucomicrobiota* (former name *Verrucomicrobia*) is a distinct phylum of the domain *Bacteria*, which was described three decades ago based on characterization of several prosthecate bacteria isolated from freshwater habitats [1]. Although only a few isolates were available at the time, molecular ecology studies revealed that these bacteria are widespread in the environment and have a much greater hidden diversity than initially expected. This was further proven by an overwhelming number of studies that detected members of *Verrucomicrobiota* (commonly addressed as verrucomicrobia) in a wide spectrum of freshwater [2,3,4], marine [5,6] and terrestrial ecosystems [7,8,9,10,11,12], as well as in the digestive tract of insects and other animals [13,14]. The relative abundance of verrucomicrobia in some of these habitats may be quite substantial. Based on 16S rRNA gene amplicon sequencing, *Verrucomicrobiota* was recognized as one of the numerically dominant phyla in soils and aquatic habitats, showing a median relative abundance of 23% in soil [8] and 9% in lake water [4]. Additional interest in these bacteria was fueled by their common evolutionary history with *Planctomycetes* and *Chlamydiae* [15], the groups of bacteria whose cell wall structure and apparent intracellular compartmentalization run contrary to the classical bacterial dogma and keep puzzling researches [16]. A real explosion of interest in verrucomicrobia, however, occurred after the discovery of probiotic properties in an intestinal anaerobe *Akkermansia muciniphila*, which resides on the mucus layer of the human intestinal tract [17,18,19,20].

Known metabolic types of these bacteria include aerobic and anaerobic chemoheterotrophs with hydrolytic potential and aerobic thermo-acidophilic methanotrophs. Most of the cultured and taxonomically characterized verrucomicrobia are chemoheterotrophs, which utilize a wide range of carbohydrates including complex natural polysaccharides, such as xylan, pectin, mannan, laminarin, cellulose, chitin, and sulphated fucoidans. The potential functional role of verrucomicrobia as polysaccharide degraders and their adaptation to carbon sources of different origins in diverse ecosystems has been demonstrated in a number of cultivation-independent studies [3,6,21,22,23,24]. Representatives of verrucomicrobia with a methanotrophic lifestyle were isolated from acidic methane-emitting geothermal habitats [25]. These bacteria are thermophilic, extremely acidophilic, autotrophic methanotrophs, which display high metabolic versatility and participate in volcanic nutrient cycles [26]. In addition to free-living members of the phylum, some symbiotic verrucomicrobia, such as the symbiont of the tunicate *Lissoclinum* sp. [27] and the marine sponge *Petrosia ficiformis* [28], have also been described.

Originally, verrucomicrobia has been divided into seven subdivisions or class-level groups based on 16S rRNA gene phylogeny [29]. Three classes that have been formally defined, *Verrucomicrobiae, Spartobacteria*, and *Opitutae*, correspond to the subdivisions 1, 2, and 4. Subdivision 3 was initially represented solely by soil-derived clone sequences but later accommodated several described isolates of soil bacteria. Members of subdivision 5 occur in various anoxic environments ranging from hypersaline sediments to wastewater and the intestine of animals. The only cultured representative was isolated from a suboxic layer of a hypersaline microbial mat, which allowed the authors of the corresponding study to categorize subdivision 5 as the novel phylum *Kiritimatiellaeota* [30]. Subdivision 6 included aerobic thermo-acidophilic methanotrophs from geothermal habitats [25], while subdivision 7 remained uncultured. *Lentisphaerae* was described as a phylum related to *Verrucomicrobiota* [15,31]. Adoption of a comparative genome-based analysis as a navigation tool in microbial systematics, however, revised the hierarchical structure of *Verrucomicrobiota*. Thus, according to the Genome Taxonomy Database version 10-RS226 (gtdb.ecogenomics.org, accessed on 10 March 2025), this phylum accommodates three classes, namely *Verrucomicrobiae*, *Lentisphaeria*, and *Kiritimatiellia*, and the candidate class CALLYA01.

In contrast to the rapidly expanding data retrieved in cultivation-independent studies, the pool of cultivated and characterized members of the *Verrucomicrobiota* remains very limited in comparison to that for other ubiquitous bacterial phyla. The apparent explanation for this phenomenon is that most verrucomicrobia are difficult to isolate and to manipulate in the laboratory. Besides specific cultivation approaches, such as extended incubation time or the use of low-nutrient media, polymeric growth substrates and gellan gum (phytagel, gelrite) instead of agar [32,33,34,35,36,37], obtaining isolates of verrucomicrobia requires a lot of patience and high skill when culturing unusual microbes.

As reported recently, a co-cultivation approach, in which aseptic duckweed is inoculated with environmental samples, co-cultivated for a specific period, with the duckweed roots then used as an isolation source, works well for laboratory cultivation of phylogenetically diverse verrucomicrobia [38,39]. The study described below also involved a co-cultivation approach, since the target *Verrucomicrobiota*-affiliated bacterium was detected in a methane-fed bioreactor running with a *Methylococcus*-dominated methane-oxidizing consortium. These cultivation experiments were focused on using fast-growing methanotrophs of the genus *Methylococcus* as potential producers of a single-cell protein from natural gas, as described in our previous publications [40,41]. The novel isolate obtained from this methane-oxidizing consortium, strain Vm1, was a member of the class *Opitutae* and affiliated with the cosmopolitan phylogenetic clade of free-living and host-associated verrucomicrobia. This study was initiated in order to characterize the physiology of this microorganism, to determine its genome-encoded potential, and to define the mechanisms behind its association with methanotrophic bacteria. We also performed a pan-genome analysis of strain Vm1 and related verrucomicrobia to gain an insight into the reasons behind the wide distribution of these bacteria in the environment.

## 2. Materials and Methods

### 2.1. Methane-Fed Bioreactor and Methane-Oxidizing Consortium Composition

**Figure 1:**
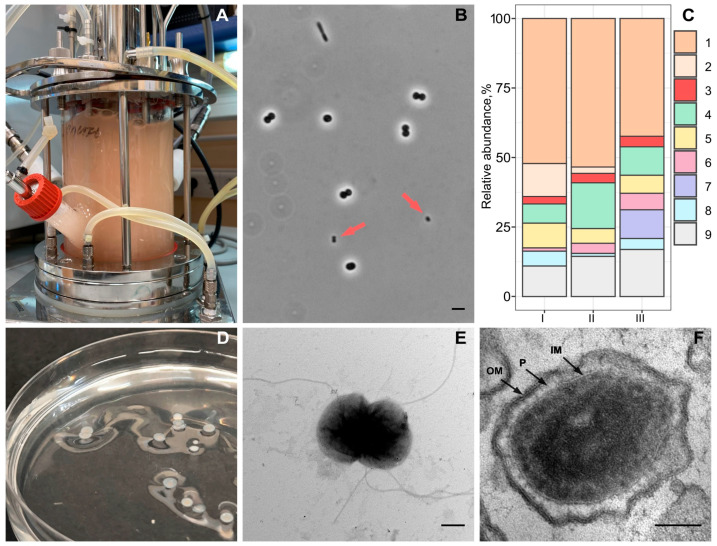
Isolation, cell morphology and ultrastructure of strain Vm1. (**A**) The methane-fed bioreactor used in this study. (**B**) A phase-contrast micrograph showing ultra-small coccoid cells of strain Vm1 (indicated by red arrows) among other bacteria in the *Methylococcus*-dominated microbial association; bar, 2 µm. (**C**) Bacterial community composition in the bioreactor after 7 (I), 14 (II) and 22 (III) days of cultivation as determined by Illumina-based 16S rRNA gene sequencing: 1—*Methylococcus*, 2—*Methylophilus*, 3—uncultured *Opitutaceae*, 4—*Brevibacillus*, 5—*Ideonella*, 6—*Allorhizobium*, 7—uncultivated *Sphingobacteriales*-affiliated bacteria, 8—uncultivated *Chitinophagales*-affiliated bacteria and 9—other minor components. (**D**) The colonies formed by strain Vm1 on mNMS medium supplemented with fructose, peptone and yeast extract and solidified with gellan gum. (**E**) Electron microscopy of contrasted cell specimens of strain Vm1 showing the presence of numerous flagella; bar, 0.2 µm. (**F**) Electron microscopy of ultrathin cell sections of strain Vm1 grown on fructose; bar, 0.1 µm.

Strain Vm1 was isolated from a 1.5 L bioreactor (GPC BIO, Perigny, France) running in continuous mode with a methane-oxidizing microbial consortium dominated with *Methylococcus* sp. Concept-8 [40] and natural gas (CH_4_, 97.3%; ethane, 1.8%; propane, 0.5%) as a growth substrate (Figure 1A). The experimental setup was described in our recent publication [41]. Briefly, the following process parameters were used: temperature, 42 °C; agitation, 1000 r.p.m.; gas flow rate, 6000 cm^3^ h^−1^; air flow rate, 18,000 cm^3^ h^−1^. The bioreactor contained 1 L of modified AMS medium (mAMS) of the following composition (g L^−1^): (NH_4_)_2_SO_4_, 0.45; MgSO_4_ × 7H_2_O, 0.125; CaCl_2_ × 2H_2_O, 0.1; and 0.1% (vol/vol) of a trace element solution described elsewhere [41]. After sterilization, 10 mL of a sterile phosphate buffer solution (a mixture of 18.9 g L^−1^ KH_2_PO_4_ and 9.6 g L^−1^ Na_2_HPO_4_ × 2H_2_O; pH 6.3) was added to the medium and cooled to 60 °C. The pH level during cultivation was kept at 5.6 via titration with 0.8% NH_4_OH solution.

The methane-oxidizing consortium developing in the bioreactor was examined daily with regard to the major cell morphotypes using phase-contrast microscopy on an Axioplan 2 microscope (Zeiss, Jena, Germany). To identify the main components of this microbial consortium, an aliquot (10 mL) of the cell suspension was sampled from the bioreactor every 10 days and taken for DNA isolation by using a DNeasy Power Soil Kit (Qiagen, Hilden, Germany). Fragments of 16S rRNA genes corresponding to the V3-V4 variable region were amplified from total DNA extracts by PCR with primers 341F (5′-CCTAYGGGDBGCWSCAG-3′) and 806R (5′-GGACTACNVGGGTHTCTAAT-3′) [42]. The PCR fragments were purified using Agencourt AMPure Beads (Beckman Coulter, Brea, CA, USA) and quantified using the Qubit dsDNA HS Assay Kit (Invitrogen, Carlsbad, CA, USA). The obtained amplicons were pooled together in equal molar amounts and sequenced on the Illumina MiSeq instrument (2 × 300 nt reads) (San Diego, CA, USA). The retrieved 16S rRNA gene sequences were analyzed with QIIME2 v.2019.10 (https://qiime2.org, accessed on 20 June 2023) [43]. Q-score and VSEARCH version 2024.10.0 uchime plugin were used for sequence quality control, denoising and chimera filtering [44,45]. Operational Taxonomic Units (OTUs) were clustered following singleton removal by applying the VSEARCH plugin [45] with open-reference function using the SILVA 138 SSU database [46] with 97% identity. Taxonomy assignment was performed using BLAST against the Silva v. 138 database with 80% identity.

### 2.2. Isolation and Identification of an Ultra-Small Bacterium

The main targets of our isolation efforts were unusual ultra-small cocci with a mean diameter of approximately 0.4 µm, which were consistently detected among other routinely observed bacterial morphotypes in the examined methane-oxidizing consortium, such as coccoid cells of *Methylococcus* sp. and rod-shaped cells of satellite heterotrophic bacteria (Figure 1B). The isolation procedure, therefore, exploited small cell sizes of the target bacteria and included multiple series of filtration via sterile 0.45 µm filter units (Millipore). Initially, an aliquot (5 mL) of the cell suspension from the bioreactor underwent pre-filtration using this filter unit, and 1 mL of the obtained filtrate was used to inoculate two versions of the liquid media, i.e., the above-described medium mAMS and the same medium but with (NH_4_)_2_SO_4_ replaced by NaNO_3_ (mNMS). Both mineral media were supplemented with 0.25 g L^−1^ fructose and 0.2 g L^−1^ yeast extract. Since better development of the target ultra-small cell bacteria was observed in mNMS medium, it was selected for further isolation work. This included several rounds of serial dilutions in the liquid medium mNMS with fructose and yeast extract, but the final round of isolation was made by using this medium supplemented with 1% (*w*/*v*) birch wood xylan (Fluka). The obtained isolate, strain Vm1, was identified by means of comparative 16S rRNA gene sequence analysis. PCR-mediated amplification of the 16S rRNA gene of this isolate was performed using primers 9f (5′-GAGTTTGATCMTGGCTCAG-3′) and 1492r (5′-ACGGYTACCTTGTTACGACTT-3′) and the reaction conditions described by [47].

### 2.3. Electron Microscopy

Regarding electron microscopy of negatively stained preparations, the cell suspension of strain Vm1 was mixed with 0.3% aqueous solution of uranyl acetate (pH 4.0). For analysis of ultrathin cell organization, cell specimens of strain Vm1 were prepared as described elsewhere [48], with the only difference being that ultrathin sections were made on an Reichert-Jung Ultracut microtome (Vienna, Austria) and were examined with a JEM-1400 transmission electron microscope (JEOL, Tokyo, Japan) at the UNIQEM Collection Core Facility, Research Center of Biotechnology of the Russian Academy of Sciences, at an accelerating voltage of 80 kV.

### 2.4. Characterization of Physiology

Physiological tests were performed on a basal medium under different conditions, including temperatures of 4–45 °C, pH 5.0–10.0 and NaCl concentrations of 0–4.0% (wt/vol). Carbon source utilization was determined using liquid mineral mNMS medium supplemented with different individual carbon sources at a concentration of 0.05% (wt/vol). The ability to grow on methanol (0.05–0.1%, vol/vol) or methane (10–30% in the gas phase) was tested as well. Nitrogen source utilization was tested by substituting NaNO_3_ in mNMS with various alternative nitrogen sources. Control incubations were run in parallel under the same conditions but without the carbon or nitrogen source, respectively. Yeast extract was added in all experiments, including control incubations, as a growth factor at a concentration of 0.005% (wt/vol). All incubations were performed under static conditions in triplicate. The growth of strain Vm1 was monitored voa nephelometry at 600 nm in an Eppendorf BioPhotometer (Hamburg, Germany). Additional biochemical tests were performed using API 20NE (bioMérieux, Marcy-l’Étoile, France) according to the manufacturer’s recommendations.

### 2.5. Co-Culture Experiments

For co-cultivation experiments, *Methylococcus* sp. Concept-8 and strain Vm1 were inoculated in 500 mL flasks containing 50 mL of mNMS medium without any organic substrates to obtain an initial OD_600_ of 0.1. The flasks were hermetically sealed, and methane was added to reach a concentration of 30% (vol/vol) in the headspace. The control incubation was represented by flasks inoculated with strain Vm1 only. The experiment was performed in triplicate. Incubation was performed at 42 °C under static conditions and with a shaker (150 rpm). After two days of incubation, 5 mL of cell suspensions from the experimental flasks was transferred to flasks with a fresh medium and a newly refilled headspace. These culture transfers were repeated five times. Aliquots of the cell suspensions were periodically sampled from experimental flasks and used for cell counting via phase-contrast microscopy.

### 2.6. Genome Sequencing and Annotation

For genome sequencing, the culture of strain Vm1 was grown in mNMS mineral medium supplemented with 0.25 g L^−1^ fructose, 0.2 g L^−1^ peptone, and 0.2 g L^−1^ yeast extract at 30 °C on a rotary shaker at 120 rpm for 2 days. The cells were harvested and the genomic DNA was extracted using the standard cetyltrimethyl ammonium bromide (CTAB) and phenol-chloroform protocol [49]. The paired-end (2 × 300 bp) genomic library was prepared using the NEBNext Ultra II DNA Library Prep Kit (New England Biolabs, Ipswich, MA, USA) and sequenced on an Illumina MiSeq platform (San Diego, CA, USA). Raw Illumina reads were processed by removing primer sequences with Cutadapt v1.17 [50] and trimming low-quality ends (Q < 30) using Sickle v1.33 (https://github.com/najoshi/sickle). For long-read sequencing, libraries were prepared with the Oxford Nanopore 1D Ligation Sequencing Kit (SQK-LSK108) (Oxford, UK) and sequenced on a MinION device (Oxford Nanopore Technologies, Oxford, UK) using R9.4 flow cells (FLO-MIN106). Short and long reads were assembled using Unicycler v.0.4.8 [51], followed by assembly refinement with Pilon v1.24 [52]. Assembly quality was assessed using Quast 5.0 [53] and Busco 5.1.2 [54]. Protein-coding sequences (CDSs) were functionally annotated with Prokka v1.14.5 [55].

### 2.7. Phylogenomic Analysis

A genome-based tree for strain Vm1, its closest phylogenetic relatives and other representative members of the phylum *Verrucomicrobiota* was reconstructed using the Genome Taxonomy Database and GTDB-toolkit (https://github.com/Ecogenomics/GtdbTk), version 09-RS220. The maximum likelihood phylogenetic tree was built using Mega X software [56]. Average nucleotide identity (ANI) values for strain Vm1 and phylogenetically closely related verrucomicrobia were estimated using the Pyani program [57].

### 2.8. Pan-Genome Analysis

The pan-genome was constructed using the anvi’o pan-genomic workflow using the MCL algorithm (Euclidean distance metric; Ward linkage) with the flags “-minbit 0.5” and “-mcl-inflation 10” [58]. Prior to pan-genome analysis, all genomes taken for the analysis were reannotated using Prokka v1.14.5. CDSs were additionally annotated via the execution of anvi’o internal programs including “anvi-run-ncbi-cogs”, “anvi-run-hmms”, “anvi-run-pfams”, and “anvi-run-kegg-kofams”. Genomes were organized and visualized based on the distribution patterns of gene clusters. Pairwise average nucleotide identity (ANI) values were calculated using the “anvi-compute-genome-similarity” program implemented in anvi’o.

### 2.9. Search for the Genome-Encoded Xylan-Degrading Enzymes

In this work, we employed the CAZy classification of glycoside hydrolases, which is based on the homology of the amino acid sequences of their catalytic domains [59]. A search for potential glycoside hydrolases among the proteins encoded in the genome of Vm1 was carried out on 25 March 2024 on the dbCAN3 server (https://bcb.unl.edu/dbCAN2/index.php) by means of three algorithms: HMMER: dbCAN, DIAMOND: CAZy, and HMMER: dbCAN-sub [60]. A list of experimentally characterized enzymes for the GH43 family of glycoside hydrolases was compiled based on the information available in the CAZy database (https://www.cazy.org/GH43_characterized.html, accessed on 7 April 2024. The amino acid sequences of GH43 family members (GenPept, XOM08569.1 and XOM10214.1) were used to search for their close homologues in the NCBI database (section “non-redundant protein sequences”) with BLASTP software (http://www.ncbi.nlm.nih.gov/, accessed on 16 April 2024). Multiple sequence alignment was performed manually using BioEdit 7.2 software (https://bioedit.software.informer.com/7.2/) by taking into account the results of pairwise alignments performed with BLASTP. The results of the multiple alignments (after removal of the most variable sequence fragments) were used to construct phylogenetic trees using the PROTPARS program (Protein Sequence Parsimony method, MP) from the PHYLIP 3.6 package (https://phylipweb.github.io/phylip/). Moreover, the programs SEQBOOT, PROTPARS, and CONSENSE were successively used to derive confidence limits, estimated by 1000 bootstrap replicates for each node. Coding densities of GH genes in *Oleiharenicola* genomes were calculated based on Prokka-annotated protein-coding gene counts.

## 3. Results

### 3.1. Methane-Oxidizing Consortium Composition in a Bioreactor

The search for the identity of ultra-small coccoid microorganisms that developed in the methane-fed bioreactor started with molecular identification of microbial community composition in cell suspensions after 7, 14 and 22 days of cultivation. A total of 24,947 partial (average length, ~260 bp) 16S rRNA gene sequences were retrieved from the examined samples of cell suspensions. The pool of *Methylococcus*-affiliated 16S rRNA gene fragments comprised 45.1–55.5% of all reads in the analyzed datasets (Figure 1C). The major groups of methanotroph-associated satellite bacteria were represented by methylotrophs of the genus *Methylophilus* (2.4–12.0% of total reads) and heterotrophs of the genera *Brevibacillus* (7.1–17.1%), *Ideonella* (5.3–9.1%) and *Allorhizobium* (1.2–6.3%) as well as uncultivated *Sphingobacteriales-* and *Chitinophagales*-affiliated bacteria (0–12 and 1.1–5.4%, respectively). Other minor groups (with a relative abundance below 5%) included representatives of the families *Commamonadaceae*, *Chitinophagaceae* and *Chromobacteriaceae* as well as the verrucomicrobial family *Opitutaceae*. The *Opitutaceae*-affiliated 16S rRNA gene sequences were detected in all examined samples of cell suspensions and were present in a relative abundance of 2.7–4.1%. Given that all cultivated and taxonomically characterized representatives of the *Opitutaceae* possess small (0.4–1.0 μm) cells of coccoid morphology, it has been hypothesized that the ultra-small cells observed in a bioreactor may belong to a member of this verrucomicrobial family.

### 3.2. Isolation and Identification of Strain Vm1

The first round of filtering cell suspensions from the bioreactor via sterile 0.45 µm filter units allowed us to reduce the number of observed microbial diversity to four to five cell morphotypes. Further rounds of serial culture dilutions in the liquid medium mNMS with fructose and yeast extract resulted in obtaining a binary culture composed of small cocci and short thin rods. The final purification step was made by replacing fructose and yeast extract in the medium mNMS with a polymeric growth substrate, xylan, which gave a clear selective advantage to the target ultra-small cocci. The latter were successfully obtained in a pure culture and the isolate was designated strain Vm1. Strain Vm1 was routinely maintained using the medium mNMS supplemented with 0.25 g L^−1^ fructose, 0.2 g L^−1^ peptone, and 0.2 g L^−1^ yeast extract (further addressed as a basal medium) and sub-cultured in one-week intervals. The standard incubation temperature was 30 °C.

On agar-solidified medium mNMS with fructose, peptone and yeast extract, strain Vm1 formed small (≤0.5 mm), circular, colorless, smooth colonies after incubation for 7 days. On mNMS medium solidified with gellan gum, growth of the isolate was accompanied by the formation of depressions around colonies (Figure 1D). The colonies were composed of small (0.35 to 0.5 μm in diameter) motile cocci that occurred singly or in pairs. Examination of negatively stained cells of strain Vm1 showed the presence of peritrichially located flagella (Figure 1E). The cell ultrastructure of strain Vm1 was examined via transmission electron microscopy. The outer membrane and the inner membrane were clearly separated with a periplasmic space and the nucleoid was concentrated in the middle of the cell (Figure 1F).

**Figure 2:**
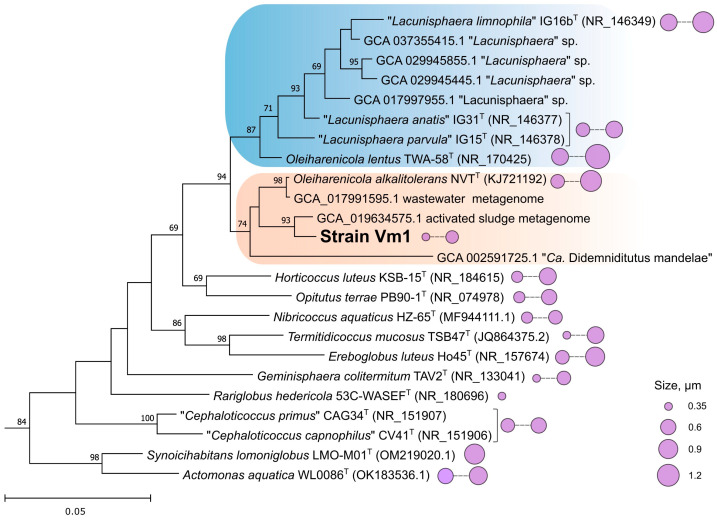
The 16S rRNA gene-based maximum likelihood tree showing the phylogenetic position of strain Vm1 in relation to described members of the verrucomicrobial family *Opitutaceae*. Bootstrap values (percentages of 1000 data resamplings) > 60% are shown. The clade defined by *Oleiharenicola alkalitolerans* NVT^T^ and *Ca*. Didemniditutus mandelae is highlighted in coral, while the clade of “*Lacunisphaera*”-related verrucomicrobia is highlighted in blue. Cell sizes of the characterized representatives are indicated by the circles on the right. The root is composed of members of the *Methylococcaceae* clade. Bar, 0.05 substitutions per nucleotide position.

Phylogenetic 16S rRNA gene-based analysis confirmed that the novel isolate affiliates with the family *Opitutaceae* and belongs to the phylogenetic clade defined by the genus *Oleiharenicola* (Figure 2). The type strain of the type species of this genus, *O. alkalitolerans* NVT^T^, was isolated from a methane-rich oilsands tailings pond in Alberta, Canada [61]. The 16S rRNA gene sequence similarity between strain Vm1 and *O. alkalitolerans* NVT^T^ was 97.90%. This clade also encompassed several MAGs (metagenome-assembled genomes) retrieved from wastewater and activated sludge as well as the symbiont of the tunicate *Lissoclinum* sp., *Candidatus* Didemniditutus mandelae [27]. The latter was only distantly related to strain Vm1 and displayed 92.44% 16S rRNA gene sequence similarity. The clade defined by *O. alkalitolerans* NVT^T^ and strain Vm1 was most closely related (95.53–96.65% 16S rRNA gene sequence similarity) to the clade of freshwater *Lacunisphaera* species [62].

Notably, the second described representative of the genus *Oleiharenicola* from irrigation water, *O. lentus* [63], clustered together with *Lacunisphaera* spp. (Figure 2), which was further confirmed by the comparative genome analysis (see below). Morphologically, all currently described members of the family *Opitutaceae* are represented by small cocci with cell sizes displayed in Figure 2. Among these, strain Vm1 represented one of the smallest morphotypes, reaching a minimal cell diameter of 0.35 μm.

### 3.3. Physiology and Growth Substrates

Strain Vm1 was capable of growth at pH values between 5.5 and 9.0 (with an optimum at pH 6.5–8.0) and at temperatures between 20 and 43 °C (with an optimum at 42 °C). The elevated optimum growth temperature is a distinctive feature of strain Vm1 compared to other members of the genera *Oleiharenicola* and *Lacunisphaera*, which generally exhibit growth optima between 25 and 30 °C and rarely tolerate temperatures above 40 °C. This characteristic of strain Vm1 may reflect its adaptation to conditions in a bioreactor, where a temperature of 42 °C supports optimal growth of the methanotrophic strain-producer.

In contrast to the closely related *O. alkalitolerans* NVT^T^, which tolerates up to 3% NaCl in the medium, the growth of strain Vm1 was inhibited by salt concentrations above 0.5% (*w*/*v*). The doubling time was 2.5 and 2.3 h under static conditions and on a shaker (150 rpm), respectively. No growth was observed under anaerobic conditions.

The new isolate was a versatile heterotroph, capable of utilizing a wide range of organic substrates. It metabolized a variety of monosaccharides, including glucose, fructose, galactose, mannose, L-rhamnose and xylose, and the disaccharides (cellobiose, lactose and maltose). D-fructose and D-glucose supported the highest growth rates, but growth on glucose induced morphological polymorphism, resulting in a significant proportion of enlarged, turgid cells. The new isolate grew well on several polysaccharides, such as xylan, starch, dextrin and locust bean gum, as well as on some organic acids (pyruvate, succinate, malate, fumarate, lactate) and some amino acids (L-alanine, L-tyrosine, L-proline, L-glutamine). Optimal growth occurred with the addition of peptone and yeast extract to the sugar substrates, presumably as sources of essential cofactors. However, in the absence of sugars, peptone and yeast extract were independently capable of supporting growth. Strain Vm1 did not grow on methane or methanol as sole carbon and energy sources, thus excluding a methanotrophic lifestyle. Enzymatic analysis of strain Vm1 using the standard API 20NE (bioMérieux) revealed the activity of several enzymes, including β-glucosidase (ESC) and β-galactosidase (PNPG). Strain Vm1 was not capable of reducing nitrates to nitrites and molecular nitrogen.

### 3.4. Growth in a Co-Culture with Methanotroph

Given that strain Vm1 was isolated from a *Methylococcus*-dominated microbial consortium, we evaluated the capacity of this methanotroph to sustain the growth of our novel verrucomicrobial isolate. For this, strain Vm1 and *Methylococcus* sp. Concept-8 were co-cultured in mNMS mineral medium with methane as the sole carbon and energy source. The obtained binary culture was then subjected to a series of five successive transfers in the same mineral medium with methane in the gas phase. Monitoring of the culture composition via phase-contrast microscopy confirmed stable development of strain Vm1 in a co-culture with methanotroph. The proportion of Vm1 cells in the co-cultures incubated under static conditions varied between 9.2% and 15.9%. The co-cultures incubated on a shaker at 150 rpm contained 5.0–12.8% cells of strain Vm1. These results confirmed that strain Vm1 is capable of stable growth in a co-culture with methanotrophic bacteria, presumably by relying on its growth-associated products.

### 3.5. Genome Analysis and Phylogenomic Reconstructions

A hybrid approach employed a combination of short-read and long-read sequencing to determine the genome sequence of strain Vm1. Illumina MiSeq short-read sequencing generated 657.2 Mb, while Oxford Nanopore sequencing yielded 375.4 Mb with a mean read length of 8455 bp. The assembled genome of strain Vm1 consisted of a 4.6 Mb chromosome and a plasmid of 143.8 kb. The total genome coverage was 216×. No evidence of contamination was detected in the final assembly. The DNA G + C content values of the chromosome and plasmid were 69.1 and 62.6%, respectively. Both sequences were finished and had no gaps. The genome sequence contained a single *rrn* operon (16S-23S-5S rRNA), 52 tRNA genes, and 4182 predicted protein-coding sequences.

**Figure 3:**
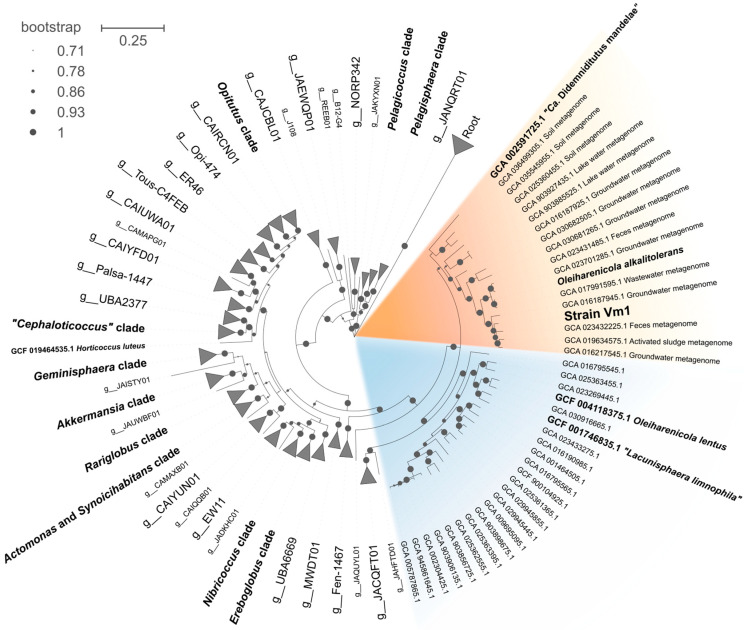
The phylogenomic maximum likelihood tree constructed for *Opitutaceae*-affiliated taxonomically described bacteria, *Candidatus* Didemniditutus mandelae and MAGs retrieved from various habitats. The tree was constructed using GTDB version 09-RS220. The genome of *Oleiharenicola alkalitolerans* NVT^T^ was imported from the IMG database. The analyzed dataset included 308 amino acid sequences. The final dataset contained 5035 positions. The clade defined by *Oleiharenicola alkalitolerans* NVT^T^, strain Vm1 and Ca. Didemniditutus mandelae is highlighted in orange, while the clade of “*Lacunisphaera*”-related verrucomicrobia is highlighted in blue. The root is composed of 18 planctomycetal genomes. Bar, 0.25 substitutions per amino acid position.

The phylogenomic analysis positioned strain Vm1 within a large well-supported clade defined by the earlier described verrucomicrobial species *Oleiharenicola alkalitolerans*, the symbiont of the tunicate *Lissoclinum* sp., *Candidatus* Didemniditutus mandelae, and numerous MAGs retrieved from various habitats (Figure 3). The average nucleotide identity (ANI) value determined for genomes of strain Vm1 and *Oleiharenicola alkalitolerans* NVT^T^ constituted 80.4%, which is well below the species-level threshold of 95%, indicating that strain Vm1 represents a novel species within the genus *Oleiharenicola*.

Strain Vm1 harbored canonical pathways of central carbohydrate metabolism, including the Embden–Meyerhof pathway, the tricarboxylic acid (TCA) cycle, the pentose phosphate pathway and gluconeogenesis. Genetic determinants of methanotrophy and methylotrophy were not identified. The genome encoded alcohol and aldehyde dehydrogenases for potential ethanol utilization.

However, growth on ethanol as well as acetate as sole carbon sources was not feasible due to the absence of genes for isocitrate lyase and malate synthase, which are required for the functioning of the glyoxylate cycle. The ethylmalonyl-CoA pathway, which could functionally substitute the glyoxylate cycle, was also not encoded [64]. The genome encoded anaplerotic reactions of the TCA cycle catalyzed by phosphoenolpyruvate carboxylase and malic enzyme, enabling malate utilization as a sole substrate. Malate can either be oxidized by malate dehydrogenase (Mdh) to oxaloacetate or by malic enzyme to pyruvate via oxidative decarboxylation. Oxaloacetate can then contribute to amino acid (aspartate and asparagine) biosynthesis or replenish the TCA cycle [65]. The genome also encoded the complete PVM-like BMC locus which encapsulates a class II aldolase as a key enzyme with the potential substrates L-rhamnose and L-fucose [66].

### 3.6. Pan-Genome Analysis of Oleiharenicola–Candidatus Didemniditutus Clade

**Figure 4:**
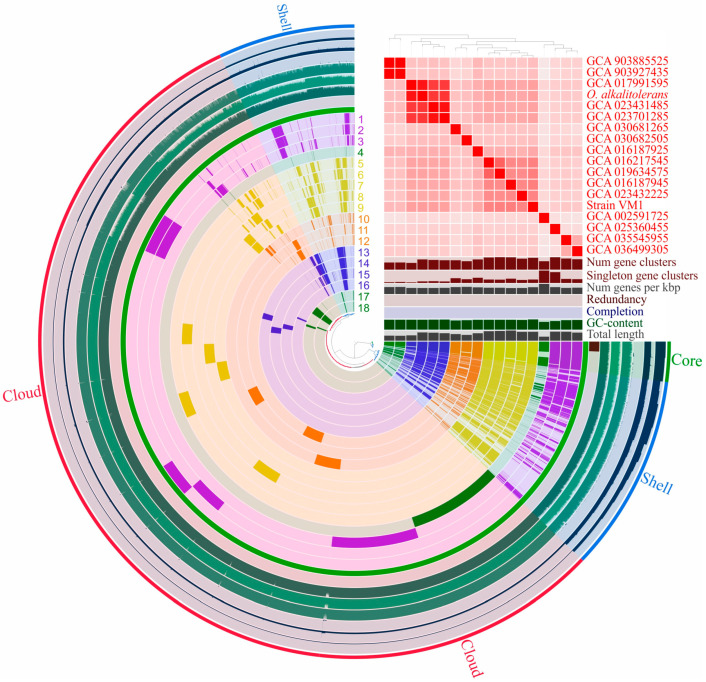
The pan-genome constructed by using the anvi’o pangenomics workflow and the pool of genomes including *Oleiharenicola alkalitolerans* NVT^T^, *Ca*. Didemniditutus mandelae, strain Vm1 and 15 good-quality (completeness 80%, no contamination) metagenome-assembled genomes affiliated with the *Oleiharenicola*–*Ca*. Didemniditutus phylogenetic clade. Clustering of genomes based on the presence/absence patterns of 19,992 pan-genomic clusters.

Only genomes with >75% completeness and <6% contamination were considered for the pan-genome analysis, resulting in 18 genomes meeting these criteria. Pan-genome, reconstructed using anvi’o [58], comprised 19,992 gene clusters, with 546 genes clusters classified as core genes (Figure 4). Genes outside the core genome but present in at least three genomes formed 4557 gene clusters, representing the shell genome. The remaining 14,888 gene clusters were part of the cloud genome, with 11,822 classified as singletons, appearing in only one genome.

**Figure 5:**
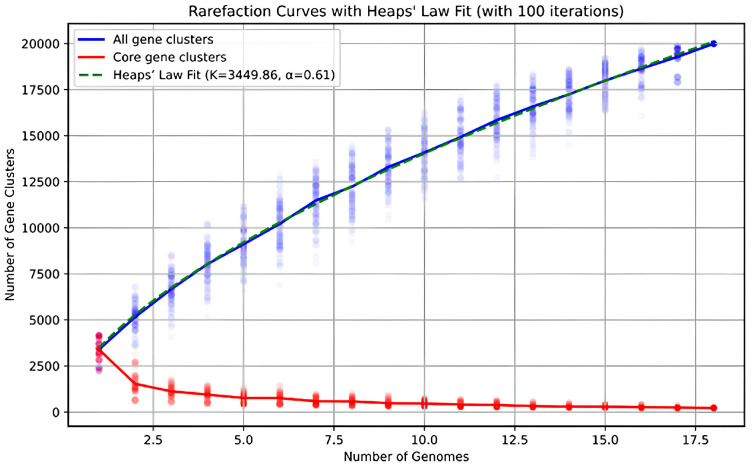
The *Oleiharenicola–Candidatus* Didemniditutus core genome (red) and pan-genome (blue) as a function of the number of genomes included.

The pan-genome of the *Oleiharenicola* group is large and open. Figure 5 depicts how the number of core genes and the total pan-genome size change as more genomes are added. Extrapolation of the pan-genome curve suggests that even with 100 sequenced genomes, the addition of another would still introduce approximately ~350 new genes. The most abundant COG categories across all fractions of the pan-genome were cell wall/membrane/envelope biogenesis (M) and the category of unknown function (S). In the core genome, the other most represented COG categories were translation, ribosomal structure and biogenesis (J); coenzyme transport and metabolism (H); energy production and conversion (C); and replication, recombination and repair (L). The shell genome was primarily enriched in amino acid metabolism and transport (E); carbohydrate metabolism and transport (G); energy production and conversion (C); and transcription (K). For the cloud genome, the dominant COG categories were signal transduction mechanisms (T); carbohydrate metabolism and transport (G); transcription (K); and replication, recombination and repair (L).

*Verrucomicrobia* is recognized for its ability to degrade complex carbohydrates, relying on diverse glycoside hydrolases (GHs) to metabolize oligo- and polysaccharides [3,26,67].

**Figure 6:**
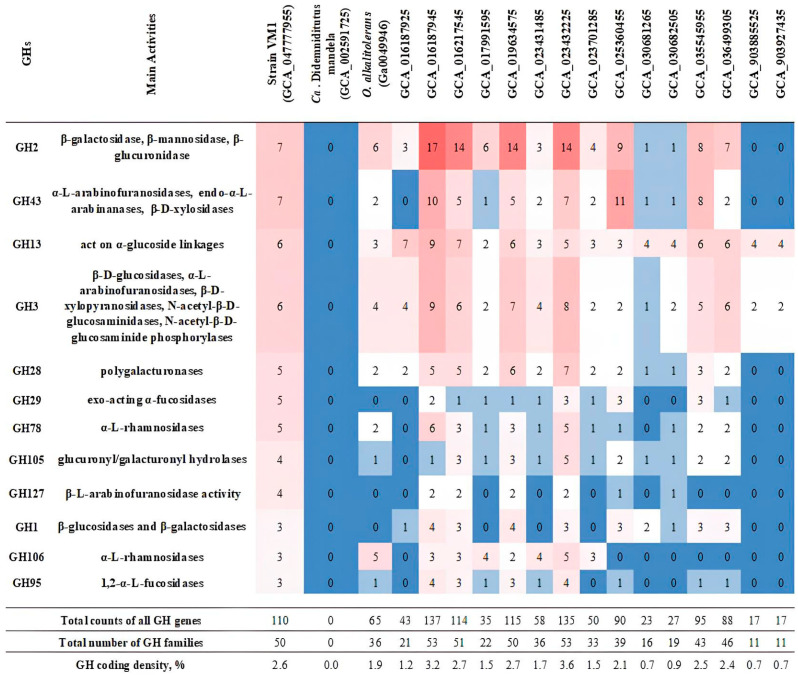
The abundance and diversity of glycoside hydrolase (GH) families across *Oleiharenicola*–*Candidatus* Didemniditutus clade genomes. The figure summarizes gene counts for the 11 most abundant GH families identified across all genomes, as well as total GH gene counts and the number of distinct GH families per genome. A heatmap was applied to visually emphasize the variation in gene abundance: counts increase from light blue to dark coral.

The pan-genome of the *Oleiharenicola*–*Candidatus* Didemniditutus clade encompassed gene clusters corresponding to 80 distinct GH families (from the CAZy classification; [59]), with substantial variation in GH content among individual genomes (11–53 families per genome, except for *Candidatus* Didemniditutus mandelae; Figure 6). This diversity likely reflects adaptation to distinct ecological niches. The GH repertoire ranged from complete absence (in the case of *Candidatus* Didemniditutus mandelae, which, however, has many GH pseudogenes) to remarkably high abundance of 137 GHs in an MAG from a groundwater planktonic microbiome (GCA_016187945). However, this variability was not strictly proportional to genome size or estimated completeness. To account for these differences, we calculated the GH coding density (i.e., the percentage of predicted protein-coding genes annotated as GHs), which ranged from 0.7% to 3.6% in GH-containing genomes. High coding densities (>2.4%) were observed in MAGs retrieved from the groundwater planktonic microbiome (GCA_016187925, GCA_016217545), soil (GCA_035545955, GCA_036499305), activated sludge (GCA_019634575) and cattle feces (GCA_023432225), as well as in the genome of strain Vm1. In contrast, MAGs derived from lake water metagenomes (GCA_903885525, GCA_903927435) and groundwater (GCA_030682505) contained fewer GH genes (<0.9%), suggesting limited carbohydrate utilization potential. The most frequently detected GH families across all genomes included GH2, GH3, GH13, GH28, GH29 and GH43 (Figure 6), with each of them associated with a broad range of activities [59]. Also, among the leaders were the families GH78 and GH106, which contain α-L-rhamnosidases (EC 3.2.1.40). The presence of multiple copies of the genes for α-L-rhamnosidase is noteworthy in connection with the ability of strain Vm1 to grow on rhamnose-containing *Methylococcus*-produced exopolysaccharides [68]. Thus, it can be inferred that strains isolated from carbohydrate-rich environments exhibit a comparatively higher genomic abundance of genes involved in the degradation of such substrates, relative to strains from other ecological niches.

### 3.7. Phylogeny of Xylan-Degrading Enzymes in Strain Vm1 and Related Verrucomicrobia

Xylan β-1,4-xylosidase (EC 3.2.1.37) is a widespread glycoside hydrolase catalyzing hydrolysis of (1→4)-β-D-xylans to remove successive D-xylose residues from the non-reducing termini [69].

In the CAZy database [59], known xylan β-1,4-xylosidases are classified into 14 glycoside hydrolase families: GH1 (2 proteins), GH2 (1), GH3 (83), GH5 (4), GH8 (0), GH10 (4), GH11 (1), GH30 (4), GH39 (20), GH43 (73), GH51 (1), GH52 (9), GH54 (1), GH116 (1) and GH120 (5). Among them, the GH43 family (clan GH-F) is functionally diverse, consisting of 39 subfamilies (GH43_1–GH43_39). Only eleven of these subfamilies (GH43_1, GH43_2, GH43_10, GH43_11, GH43_12, GH43_14, GH43_16, GH43_22, GH43_27, GH43_29 and GH43_35) are known to include xylan β-1,4-xylosidases.

Strain Vm1 encoded a broad set of glycoside hydrolases, totaling 110 GH proteins from 50 different GH families (Figure 6), suggesting the ability to utilize a wide range of oligo- and polysaccharides. The overall GH gene density, normalized by total gene count, was estimated at 2.63%. Among the identified GHs, seven belonged to the GH43 family, including three proteins from subfamilies GH43_2 (GenPept, XOM11226.1) and GH43_10 (XOM08569.1 and XOM10214.1). Genome annotation of strain Vm1 with Prokka predicted three potential xylan 1,4-β-xylosidases (EC 3.2.1.37): the two GH43_10 members mentioned above and one GH39 family protein (XOM11214.1). We focused on phylogenetic analysis of the two GH43_10 proteins and their closest homologues.

We first revisited our earlier published work on pairwise sequence comparisons among GH43 family proteins [70,71], in which seven distinct subfamilies (43a–43g) were initially identified. Proteins now assigned to the modern GH43_10 subfamily correspond to the earlier 43c group. We checked all 43c-proteins known at that time and reclassified them into four currently recognized subfamilies (GH43_9–GH43_12). Upon reclassification, we identified four proteins within GH43_10 and used them in further phylogenetic analysis.

**Figure 7:**
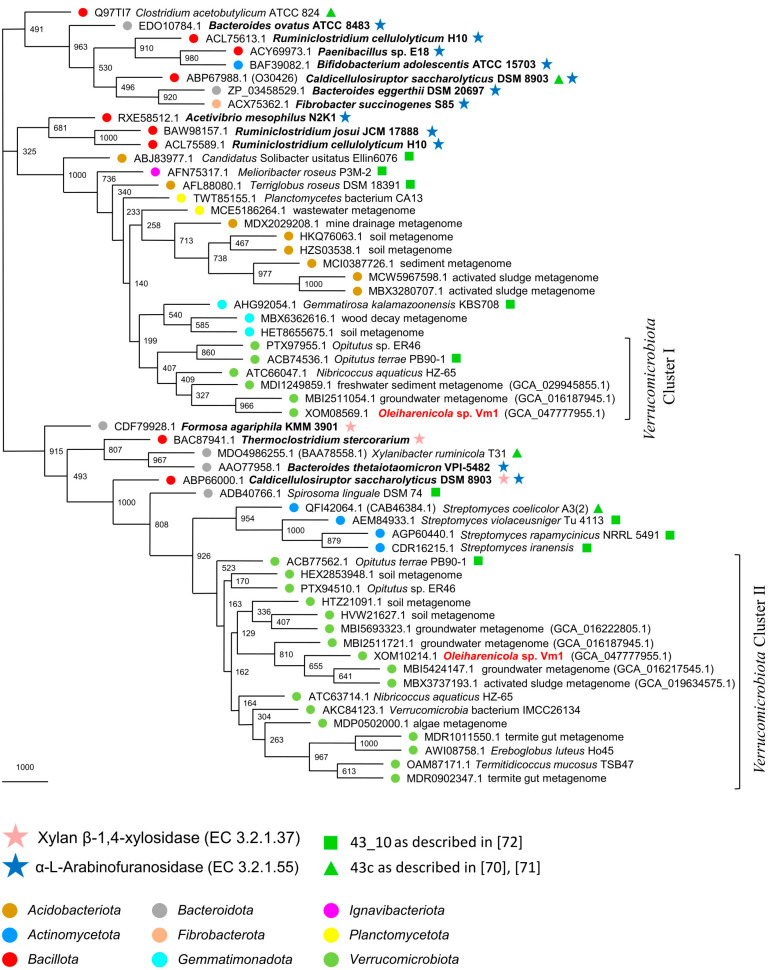
The maximum parsimony phylogenetic tree of the glycoside hydrolase subfamily GH43_10. Statistical significance of tree nodes was assessed via bootstrap analysis; the number of supporting pseudoreplicas out of 1000 is indicated at each node. Phylogenetic affiliation of proteins is indicated by colored circles. The location of 14 enzymatically characterized proteins (xylan β-1,4-xylosidases and α-L-arabinofuranosidases) is indicated by stars. The fifteen closest homologues for proteins XOM08569.1 and XOM10214.1 according to the blast search were used for analysis. All GH43_10 subfamily members analyzed in [70] (BAA78558.1, CAB46384.1, and O30426) and [71] (O30426 and Q97TI7) were used as well. The five closest homologues for the proteins XOM08569.1 and XOM10214.1 from [72] list were also used for analysis. A total of 58 proteins were analyzed.

In the original description of GH43 subfamilies (GH43_1–GH43_37), phylogenetic trees were built for each subfamily (see Supplementary Materials in [72]). Specifically, the GH43_10 tree included 243 proteins. We screened this set using BLASTP with XOM08569.1 and XOM10214.1 as queries and selected the top five hits for each. We also performed a broader search in GenPept, selecting the top 15 hits for each protein. All experimentally characterized GH43_10 enzymes from the CAZy database [59] were also included. In total, 58 proteins were used in the phylogenetic analysis. The resulting MP phylogenetic tree revealed two distinct clusters of verrucomicrobial proteins (Figure 7).

Cluster I contains XOM08569.1 and its five closest homologues from GenPept. It is part of a larger, taxonomically diverse and highly supported clade (100% bootstrap), situated near a group of experimentally characterized α-L-arabinofuranosidases. This strongly suggests that all members of Cluster I likely share the same enzymatic activity.

Cluster II includes XOM10214.1, its fifteen closest homologues from GenPept and a protein from *Opitutus terrae* PB90-1 (ACB77562.1). It is also part of a larger, stable clade (91.5% bootstrap support) that contains four experimentally verified enzymes: two xylan β-1,4-xylosidases (BAC87941.1 and CDF79928.1), one α-L-arabinofuranosidase (AAO77958.1) and one bifunctional enzyme with both activities (ABP66000.1). These data suggest that Cluster II proteins, including XOM10214.1, are bifunctional β-xylosidases/α-L-arabinofuranosidases.

All ten reference proteins that we selected from the phylogenetic tree of [72] are located within these two major clades (91.5% and 100%). Notably, *Opitutus terrae* PB90-1 contributes two proteins, ACB74536.1 and ACB77562.1, to Clusters I and II, respectively. This confirms the approximate placement of XOM08569.1 and XOM10214.1 within the broader phylogenetic landscape [72]. The high representation of verrucomicrobial proteins in Cluster II points to a long evolutionary history of these genes within *Verrucomicrobiota*, whereas the more taxonomically diverse composition of Cluster I suggests that its genes were likely acquired more recently via lateral gene transfer from other bacterial phyla.

## 4. Discussion

Strain Vm1 described in this study is an example of the ubiquitous but poorly studied verrucomicrobia that inhabits a wide variety of aquatic and soil habitats. As shown in Figure 2, cell sizes of this bacterium place it among the smallest described members of the family *Opitutaceae*. The cell volume of strain Vm1 is in the range of 0.022–0.065 µm^3^, which is far below 0.1 µm^3^, and allows us to place it in the category of ultra-small bacteria or ultramicrobacteria [73,74]. Notably, members of the verrucomicrobial genus *Opitutus* were among the first ultramicrobacteria isolated and described from a soil habitat [32,75]. The smallest described representative of the family *Opitutaceae*, *Rariglobus hedericola*, with a mean cell diameter of 0.35 µm, was isolated from a freshwater ditch in Austria [76]. These free-living verrucomicrobia maintain ultra-small cell sizes throughout their life cycle, regardless of nutrient availability in laboratory media. Interestingly, despite their small cell size, their genomes range from 4 to 6 Mb, exceeding the 3.2 Mb upper limit typically associated with classical ultramicrobacteria. [77]. The only reported example of an *Opitutaceae*-affiliated genome-reduced verrucomicrobium is a bacterial symbiont associated with the tunicate *Lissoclinum* sp., *Candidatus* Didemnitutus mandela [27]. This name was later corrected to *Candidatus* Didemniditutus mandelae before including it in the Candidatus list no. 3 [78]. The genome assembly of this symbiont is 2.17 Mb in length and estimated to be 94.2% complete. Although the genome showed signs of ongoing degradation, with numerous pseudogenes and low coding density, it contained seven copies of a polyketide synthase pathway for the mandelalides, cytotoxic compounds that provide a chemical defense for the host. This suggests a strong selective pressure on the symbiont to enhance mandelalide production [27].

As revealed by our phylogenomic analysis (Figure 3), strain Vm1 belongs to the large *Opitutaceae*-affiliated clade, which contains the genomes of *Oleiharenicola alkalitolerans* NVT^T^ and *Candidatus* Didemniditutus mandelae as well as two dozen MAGs retrieved from aquatic and soil habitats all over the world. In versions 09-RS220 and 10-RS226 of GTDB, however, this genus-level clade is defined as g_*Didemniditutus* and not as g_*Oleiharenicola* (as it should be). The reason is that the genome sequence of *Oleiharenicola alkalitolerans* NVT^T^ has been deposited in the IMG database only and is not available in the GenBank, which serves as a source of all genomes in the GTDB. This is one of the as-yet-unsolved issues of the GTDB that remains unnoticed by many researchers. Nevertheless, comparative genomic analysis clearly places strain Vm1 within the genus *Oleiharenicola* and show that it displays the highest phylogenetic relatedness to *Oleiharenicola alkalitolerans* NVT^T^. The earlier described second species of this genus, *Oleiharenicola lentus* [63], appears to be misclassified since it affiliates with the genus “*Lacunisphaera*” [62]. The close phylogenetic relation of the type strain of *Oleiharenicola lentus*, TWA-58^T^, with representatives of the genus “*Lacunisphaera*” has been noticed before [63], but the authors choose to describe this bacterium as belonging to the genus *Oleiharenicola*, since the name *“Lacunisphaera”* has not been validly published.

Strain Vm1 displayed a number of features that clearly differentiate it from *O. alkalitolerans* NVT^T^. The cells of strain Vm1 were smaller (0.35 µm) than those of *O. alkalitolerans* NVT^T^ (0.5–1.0 µm). The isolate from the bioreactor was represented by thermotolerant bacteria with an optimum growth temperature of 42 °C, while *O. alkalitolerans* NVT^T^ did not grow at temperatures above 40 °C and grew optimally at 25–37 °C. Notably, strain Vm1 exhibited a high genomic GC content (~69%), the highest within the *Oleiharenicola*–*Candidatus* Didemniditutus clade. This aligns well with known adaptations of thermotolerant and thermophilic bacteria, whose elevated GC content enhances DNA stability at high temperatures [79]. In addition, strain Vm1 displayed a wider substrate utilization spectrum than *O. alkalitolerans* NVT^T^ and utilized L-fucose, L-rhamnose, mannose, maltose, cellobiose abd starch, but was not capable of growing on acetate and polyhydric alcohols (D-mannitol, D-sorbitol). The ANI value determined for genomes of strain Vm1 and *Oleiharenicola alkalitolerans* NVT^T^ was 80.4%, which is well below the species-level threshold of 95%. Taken together, these differences indicate that strain Vm1 represents a novel species within the genus *Oleiharenicola*. which remains to be taxonomically described.

Both *Oleiharenicola alkalitolerans* NVT^T^ and strain Vm1 were isolated from methane-rich habitats, i.e., oilsands tailings pond in northern Alberta, Canada [61] and a laboratory methane-fed bioreactor (this study), respectively. Neither of them, however, were able to grow on methane, and no genetic determinants of methanotrophy or methylotrophy were revealed in their genomes. As suggested by the results of our co-culture experiments, strain Vm1 was able to thrive in association with *Methylococcus* sp. presumably by relying on its growth-associated products, such as organic acids excreted by the methanotroph (succinate, malate) and its exopolysaccharides. The analysis of exopolysaccharides produced by *Methylococcus capsulatus* identified L-rhamnose as one of the components [68] which could be utilized by strain Vm1-like verrucomicrobia. Oilsands tailings pond in northern Alberta was also colonized with a highly active methanotrophic community dominated by *Methylocaldum* and *Methylomonas* species, which served as primary producers of organic matter from methane [80]. Other as-yet-uncultivated members of the phylogenetic clade defined by the genus *Oleiharenicola* were earlier detected by means of cultivation-independent approaches in microbial consortia with various methanotrophic bacteria that developed under oxygen-limited conditions [81,82]. Together, these findings suggest that many members of this clade may naturally associate with methanotrophic bacteria in trophic relationships. As stated above, the occurrence of multiple copies of genes encoding α-L-rhamnosidases from the families GH78 and GH106 in the genomes of strain Vm1, *Oleiharenicola alkalitolerans* NVT^T^ and several environmental MAGs (Figure 6) aligns well with the ability of these verrucomicrobia to grow on rhamnose-containing exopolysaccharides.

Pan-genome analysis revealed the differences in GH abundance and diversity within the *Oleiharenicola*–*Candidatus* Didemniditutus clade, underscoring ecological divergence and potential specialization to habitat-specific carbohydrate sources. However, we acknowledge limitations related to the incompleteness of most currently available genomes in this clade. While MAGs provide broad functional insights, they may lack resolution for certain niche-specific genes. The open nature of the pan-genome suggests that additional genomes will refine core/shell gene estimates as well as trends in GH diversity.

The presence of extensive gene repertoires for glycoside hydrolases, including α-L-rhamnosidases, in the genomes of free-living *Oleiharenicola*-related verrucomicrobia likely underpins their capacity to thrive in a wide array of environments, where complex carbohydrates are available. The special focus in our study was on xylan, one of the major plant-derived biopolymers, whose presence in the growth medium allowed for selective enrichment and isolation of the target verrucomicrobium in pure culture. Phylogenetic analysis of the potential xylan 1,4-β-xylosidases/α-L-arabinofuranosidases of strain Vm1 revealed two clades of these enzymes in the subfamily GH43_10. Notably, one of these clades was composed exclusively of the proteins from representatives of *Verrucomicrobiota*, thus indicating a long-term evolution of the corresponding genes in members of this bacterial phylum. Altogether, the variation in GH abundance and diversity within the *Oleiharenicola* clade reflects prolonged adaptation to complex carbohydrates and highlights ecological divergence and potential niche specialization.

## 5. Conclusions and Future Research Directions

The novel bacterium described in our study is one of the smallest currently characterized members of the verrucomicrobial family *Opitutaceae*. It is also the second cultivated representative of the poorly studied but globally distributed phylogenetic clade defined by the free-living *Oleiharenicola alkalitolerans* and the symbiotic *Candidatus* Didemniditutus mandelae. Recovery of strain Vm1 from the relatively simple *Methylococcus*-dominated microbial consortium of a methane-fed bioreactor suggests the potential to shed some light on the physiology and metabolic potential of these bacteria. Apparently, many representatives of the *Oleiharenicola*–*Candidatus* Didemniditutus clade may occur in nature in trophic associations with methanotrophic bacteria, thus participating in the cycling of methane-derived carbon. A promising future research direction, therefore, is the screening of laboratory and environmental methane-oxidizing consortia for the presence of Vm1-related verrucomicrobia. The availability of additional isolates of these bacteria may open the possibility of describing a novel species of the genus *Oleiharenicola* based on the variety of strains isolated from different sources. Future studies integrating substrate utilization assays can link the genomic potential of *Oleiharenicola*-related verrucomicrobia with their in situ functional roles in carbon cycling.

## Data Availability

The 16S rRNA gene sequence of strain Vm1 has been deposited in NCBI GenBank under the accession number PP889714. The assembled genome sequence of this bacterium has been deposited in NCBI GenBank under the accession numbers CP180537.1 and CP180536.1 for the chromosome and plasmid, respectively.
